# Supplementary material for: The Discrimination and Characterization of Volatile Organic Compounds in Different Areas of Zanthoxylum bungeanum Pericarps and Leaves by HS-GC-IMS and HS-SPME-GC-MS
Source: Foods. 2022 Nov 21;11(22):3745. doi: 10.3390/foods11223745 (PMC9689319; doi:10.3390/foods11223745)
Supplement: Supplementary file 1 [file foods-11-03745-s001.zip › Table S1.pdf]

Table S1. Detailed information of all the samples.

	Origin	Batch number		Origin	Batch number
SHJ1	Hancheng, Shannxi, China	202007301001	SJ1	Maoxian, Sichuan, China	202007302001
SHJ2	Hancheng, Shannxi, China	202007301002	SJ2	Maoxian, Sichuan, China	202007302002
SHJ3	Hancheng, Shannxi, China	202007301003	SJ3	Maoxian, Sichuan, China	202007302003
SHJ4	Hancheng, Shannxi, China	202007301004	SJ4	Maoxian, Sichuan, China	202007302004
SHJ5	Hancheng, Shannxi, China	202007301005	SJ5	Maoxian, Sichuan, China	202007302005
SHJ6	Hancheng, Shannxi, China	202007301006	SJ6	Maoxian, Sichuan, China	202007302006
SHJY1	Hancheng, Shannxi, China	202007301101	SJY1	Maoxian, Sichuan, China	202007302101
SHJY2	Hancheng, Shannxi, China	202007301102	SJY2	Maoxian, Sichuan, China	202007302102
SHJY3	Hancheng, Shannxi, China	202007301103	SJY3	Maoxian, Sichuan, China	202007302103
SHJY4	Hancheng, Shannxi, China	202007301104	SJY4	Maoxian, Sichuan, China	202007302104
SHJY5	Hancheng, Shannxi, China	202007301105	SJY5	Maoxian, Sichuan, China	202007302105
SHJY6	Hancheng, Shannxi, China	202007301106	SJY6	Maoxian, Sichuan, China	202007302106
