# Supplementary material for: The Discrimination and Characterization of Volatile Organic Compounds in Different Areas of Zanthoxylum bungeanum Pericarps and Leaves by HS-GC-IMS and HS-SPME-GC-MS
Source: Foods. 2022 Nov 21;11(22):3745. doi: 10.3390/foods11223745 (PMC9689319; doi:10.3390/foods11223745)
Supplement: Supplementary file 1 [file foods-11-03745-s001.zip › Table S2 .pdf]

Table S2. Reproducibility.

	Ingredients	Peak area						SD	Average value	RSD
HS-SPME-GC-MS	α-Thujene	255609091	251119081	245497443	242395244	242633284	262933508	8153010	215477237	3.78%
	Sabinene	335111735	329904053	317248321	301984044	301493178	336233231	15899507	276839153	5.74%
	Myrcene	255165365	252947601	236982620	225789060	230209760	248741438	12367875	208886245	5.92%
	Limonene	950798406	953636571	904113503	777755354	780156593	904722789	80046429	764461378	10.47%
	γ-Terpinene	283516364	276981098	269480052	262194074	263826941	302902074	15238598	239162743	6.37%
	Linalool	1155348010	1134189372	1101812909	1126068108	1126037418	1079940097	26297287	964241886	2.73%
	L-Menthol	218817611	208942347	204014592	203238657	218532148	220357540	7852017	183107845	4.29%
	γ-Terpineol	911458474	922719525	911417656	860773590	899666193	908396175	21690748	776588909	2.79%
	Linalyl acetate	6750780263	6744463121	6471706326	6487839687	6792584564	6638973413	139703559	5718007276	2.44%
HS-GC-IMS	(E,E)-2,4-Hexadienal	14517	13921	13314	13427	13783	13595	433	11856	3.65%
	Acetone	7790	7027	7304	7174	6879	7220	312	6244	4.99%
	α-Pinene	15901	14909	17131	15697	15369	17146	925	13868	6.67%
	Nerol	20470	20077	19957	20189	20165	19905	202	17281	1.17%
	4-Terpineol	7406	6954	7475	6819	6944	6998	272	6124	4.44%
	1,8-Cineole	9423	9072	8730	8855	8842	8926	247	7728	3.20%
