# Supplementary material for: The Discrimination and Characterization of Volatile Organic Compounds in Different Areas of Zanthoxylum bungeanum Pericarps and Leaves by HS-GC-IMS and HS-SPME-GC-MS
Source: Foods. 2022 Nov 21;11(22):3745. doi: 10.3390/foods11223745 (PMC9689319; doi:10.3390/foods11223745)
Supplement: Supplementary file 1 [file foods-11-03745-s001.zip › Table S3.pdf]

Table S3. Composition detected by HS-SPME-GC-MS

Count	Formula	Rt	RIRef	RIExp	Compound	CAS
1	$C_2H_4O_2$	1.67	610	-	Acetic Acid	64-19-7
2	$C_{10}H_{16}$	5.13	929	921	α-Thujene	2867-05-2
3	$C_{10}H_{16}$	5.26	937	929	2-Pinene	80-56-8
4	$C_{10}H_{16}$	6.01	974	970	Sabinene	3387-41-5
5	$C_{10}H_{16}$	6.10	979	975	β-Pinene	127-91-3
6	$C_{10}H_{16}$	6.35	991	987	Myrcene	123-35-3
7	$C_{10}H_{16}$	6.70	1005	1003	α-Phellandrene	99-83-2
8	$C_{10}H_{16}$	6.98	1017	1015	α-Terpinene	99-86-5
9	$C_{10}H_{14}$	7.19	1025	1023	P-Cymene	99-87-6
10	$C_{10}H_{16}$	7.29	1030	1027	Limonene	138-86-3
11	$C_{10}H_{18}O$	7.36	1032	1030	1,8-Cineole	470-82-6
12	$C_{10}H_{16}$	7.49	1037	1035	Ocimene	13877-91-3
13	$C_{10}H_{16}$	7.78	1049	1046	(E)-B-Ocimene	3779-61-1
14	$C_{10}H_{16}$	8.11	1060	1058	γ-Terpinene	99-85-4
15	$C_{10}H_{18}O$	8.35	1070	1066	Trans-Sabinene hydrate	17699-16-0
16	$C_{10}H_{16}$	9.02	1088	1088	Terpinolene	586-62-9
17	$C_{10}H_{18}O$	9.44	1099	1101	Linalool	78-70-6
18	$C_{10}H_{16}O$	11.01	1144	1145	Trans-Verbenol	1820-09-3
19	$C_{10}H_{18}O$	11.32	1152	1153	(+)-Citronellal	2385-77-5
20	$C_{10}H_{20}O$	12.09	1175	1172	L-Menthol	2216-51-5
21	$C_{10}H_{18}O$	12.29	1177	1177	4-Terpineol	562-74-3
22	$C_9H_{14}O$	12.73	1184	1184	4-Isopropylcyclohex-2-en-1-one	500-02-7
23	$C_{10}H_{18}O$	12.86	1189	1190	α-Terpineol	98-55-5
24	$C_{10}H_{18}O$	14.27	1197	1222	γ-Terpineol	586-81-2
25	$C_{10}H_{12}O$	15.10	1239	1241	Cuminaldehyde	122-03-2
26	$C_{10}H_{14}O$	15.29	1242	1245	Carvone	99-49-0
27	$C_{10}H_{16}O$	15.72	1253	1254	Piperitone	89-81-6
28	$C_{12}H_{20}O_2$	15.95	1257	1258	Linalyl acetate	115-95-7
29	$C_{12}H_{20}O_2$	17.34	1285	1286	Bornyl acetate	76-49-3
30	$C_{10}H_{14}O$	17.64	1289	1291	Cumic alcohol	536-60-7
31	$C_{12}H_{20}O_2$	18.05	1301	1298	4-Terpinenyl acetate	4821-04-9
32	$C_{12}H_{20}O_2$	18.98	1315	1318	Terpineol, δ- acetate	93836-50-1
33	$C_{12}H_{20}O_3$	20.22	1344	1342	Exo-2-Hydroxycineole acetate	57709-95-2
34	$C_{12}H_{20}O_2$	20.63	1350	1350	Terpinyl acetate	80-26-2
35	$C_{12}H_{20}O_2$	21.56	1364	1367	Neryl acetate	141-12-8

36	$C_{15}H_{24}$	21.98	1376	1374	(-)-α-Copaene	3856-25-5
37	$C_{12}H_{20}O_2$	22.58	1382	1385	Geranyl acetate	105-87-3
38	$C_{15}H_{24}$	22.77	1389	1388	Cubebene	13744-15-5
39	$C_{15}H_{24}$	22.93	1391	1391	β-Elemene	515-13-9
40	$C_{15}H_{24}$	24.24	1419	1415	β-Caryophyllene	87-44-5
41	$C_{15}H_{24}$	26.09	1454	1449	α-Caryophyllene	6753-98-6
42	$C_{15}H_{24}$	26.49	1461	1457	(-)-Alloaromadendrene	25246-27-9
43	$C_{15}H_{24}$	27.47	1477	1474	γ-Muurolene	30021-74-0
44	$C_{15}H_{24}$	27.62	1481	1476	Germacrene D	23986-74-5
45	$C_{15}H_{24}$	27.89	1486	1481	β-Selinene	17066-67-0
46	$C_{13}H_{20}O$	28.01	1486	1483	β-Lonone	79-77-6
47	$C_{15}H_{26}O$	28.48	1493	1491	Epicubebol	38230-60-3
48	$C_{15}H_{24}$	28.76	1499	1495	α-Muurolene	10208-80-7
49	$C_{15}H_{24}$	29.48	1513	1508	γ-Cadinene	39029-41-9
50	$C_{15}H_{24}$	30.09	1518	1520	β-Cadinene	523-47-7
51	$C_{15}H_{26}O$	30.49	1515	1528	Cubebol	23445-02-5
52	$C_{15}H_{24}$	30.77	1538	1533	α-Cadinene	24406-05-1
53	$C_{15}H_{26}O$	31.45	1549	1546	Elemol	639-99-6
54	$C_{15}H_{24}$	31.72	1557	1551	(1E,4E)-Germacrene B	15423-57-1
55	$C_{15}H_{24}O$	32.84	1576	1571	Spathulenol	6750-60-3
56	$C_{15}H_{24}O$	33.10	1581	1575	Caryophyllene oxide	1139-30-6
57	$C_{15}H_{24}O$	34.53	1606	1600	(-)-Humulene epoxide II	19888-34-7
58	$C_{15}H_{26}O$	36.33	1640	1635	T-Cadinol	5937-11-1
59	$C_{15}H_{26}O$	37.05	1653	1648	α-Cadinol	481-34-5
60	$C_{15}H_{26}O$	38.84	1684	1681	α-Bisabolol	515-69-5
61	$C_{17}H_{28}O_2$	43.23	1843	1816	Farnesyl acetate	4128-17-0
62	$C_{20}H_{32}$	44.40	1960	1952	M-Camphorene	20016-73-3
