# Supplementary figures and images for: The Discrimination and Characterization of Volatile Organic Compounds in Different Areas of Zanthoxylum bungeanum Pericarps and Leaves by HS-GC-IMS and HS-SPME-GC-MS
Source: Foods. 2022 Nov 21;11(22):3745. doi: 10.3390/foods11223745 (PMC9689319; doi:10.3390/foods11223745)

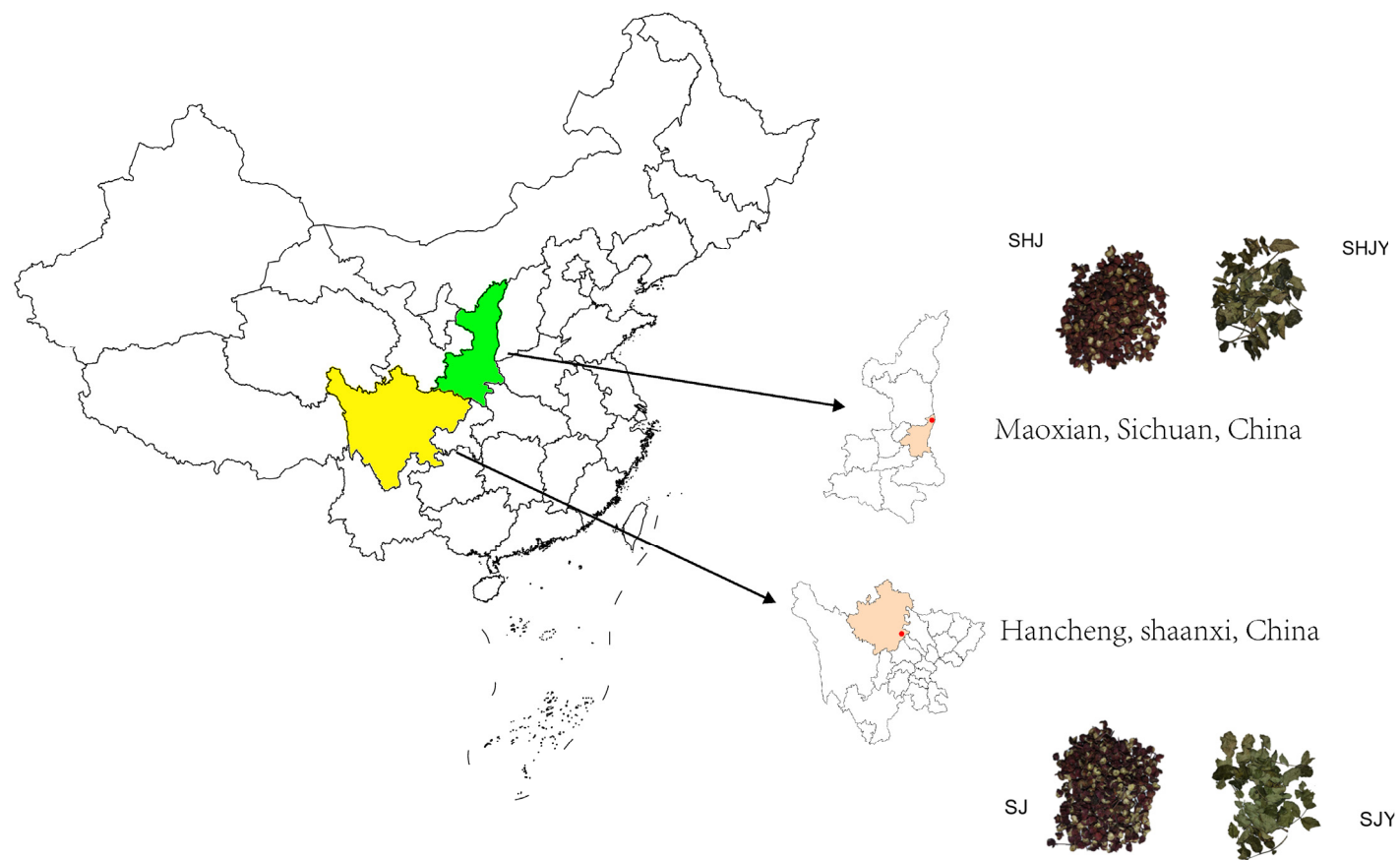

**Figure S1.** Different area of *Zanthoxylum bungeanum* pericarps and leaves.

Supplement: Supplementary file 1 [file foods-11-03745-s001.zip › Figure S1.pdf]
